# Deafness after COVID-19?

**DOI:** 10.1007/s00106-021-01041-0

**Published:** 2021-05-21

**Authors:** K. Gerstacker, I. Speck, S. Riemann, A. Aschendorff, A. Knopf, S. Arndt

**Affiliations:** Klinik für Hals‑, Nasen- und Ohrenheilkunde, Universitätsklinikum Freiburg, Killianstraße 5, 79106 Freiburg, Germany

**Keywords:** Coronavirus infections, Hearing loss, Hearing aids, Cochlear implantation, Cochlear implants

## Abstract

This article presents a case of sudden bilateral deafness in the context of a severe acute respiratory syndrome coronavirus 2 (SARS-CoV2) infection and resultant coronavirus disease 2019 (COVID-19). After treatment in the intensive care unit for acute respiratory distress syndrome and acute kidney failure, hearing ability had drastically changed. While hearing had been subjectively normal before the infection, deafness was now measured on the left and profound hearing loss on the right ear. The patient was treated with cochlea implants on the left and a hearing aid in the right ear. The hearing loss is most likely a complication of COVID-19.

Since December 2019, the coronavirus disease 2019 (COVID-19) pandemic has changed the world and the health system. At the time of writing, more than 103,377,424 cases of infection d been confirmed worldwide, with many people battling the sequelae months after recovering from a COVID-19 infection. The long-term sequelae of COVID-19 have hardly been investigated thus far.

## Case report

### Anamnesis

After contracting COVID-19 in early April, a 38-year-old man initially showed symptoms of a respiratory infection, including subjectively reduced olfactory function. In the course of the infection, there was rapidly increasing dyspnea. Mechanical ventilation was required 6 days after the initial symptoms as well as veno-venous extracorporeal membrane oxygenation (ECMO), because of acute lung failure. The transfer to our university hospital took place 5 days later. On the basis of suspected septicemia due to a bacterial superinfection, antibiotic infusion therapy was initiated using amoxicillin, which was then switched to meropenem. Furthermore, dialysis was necessary because of acute renal failure. After another 6 weeks of intensive therapy and prolonged sleep–awakening, the patient complained of hearing loss in the right ear, deafness and tinnitus in the left ear, as well as rotatory vertigo. The presence of other symptoms, such as otalgia, otorrhea, and vertigo, was denied. The patient’s pre-existing conditions were morbid obesity and arterial hypertension.

### Clinical findings

Otoscopy was unremarkable on both sides. Results of the tuning fork test (Weber/Rinne) could not be obtained, since the patient was unable to hear the tones. Spontaneous or provocation nystagmus was not present.

### Diagnosis

**Fig. 1:**
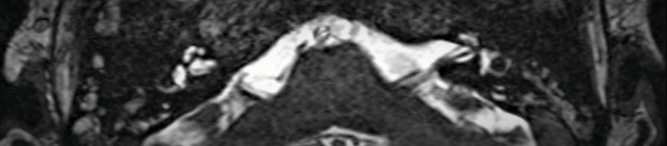
Preoperative axial magnetic resonance imaging (T2 CISS3D): no signs of cochlear obliteration

The first pure-tone audiometry after 45 days of intensive care in Freiburg at the bedside revealed an air conduction threshold on the right side of 70 dB between 1 kHz and 4 kHz. On the left side there was no air conduction threshold. Measurement of otoacoustic emissions (OAE) did not show reproducible potentials on both sides. The diagnostic tests were completed using brainstem audiometry (BERA), videonystagmography (VNG), computed tomography (CT), and magnet resonance tomography (MRI). The BERA showed no potentials on the left side. On the right side the threshold for Jewett V was 70 dB with normal interpeak latency; VNG demonstrated under-excitability on the left side. The MRI findings were unremarkable with no signs of an inflammatory process in the cochlea (Fig. 1).

The diagnoses of acute deafness in the left ear and profound hearing loss in the right ear were made.

### Treatment course

Furosemide (2 × 20 mg/day), which was applied to stimulate self-diuresis 2 days before the appearance of hearing loss, was immediately withdrawn.

**Fig. 2:**
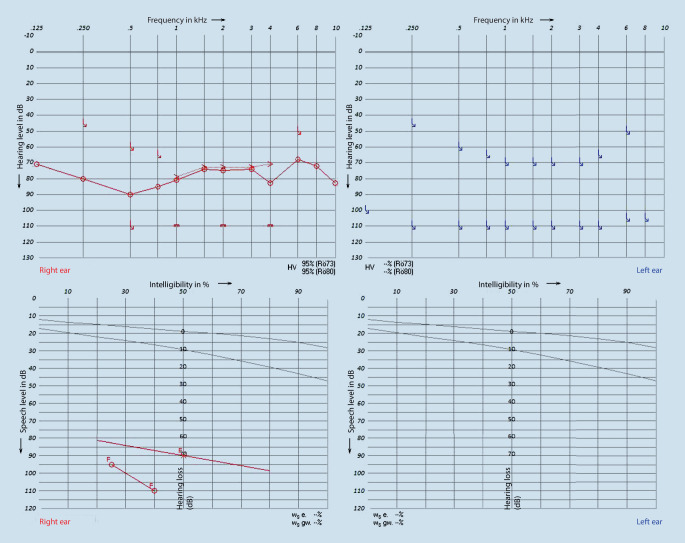
Preoperative pure-tone audiometry and Freiburg Speech Test after 53 days of intensive care and 1 week of steroid therapy

After 46 days of intensive care, directly after the complaint was reported and the diagnosis was made, bilateral intratympanic cortisone therapy (Fortecortin 4 mg) and systemic cortisone administration (prednisolone 250 mg) over 3 days were initiated. Audiometric follow-up 1 week after initiation of therapy showed no improvement in hearing (Fig. 2).

Cochlea implant (CI) surgery on the left side was indicated and performed without complications. Due to profound hearing loss in the right ear, a hearing aid was fitted. The hearing rehabilitation with CI and hearing aid restored the patient’s communication ability, which was necessary for the urgently needed neurological rehabilitation.

**Fig. 3:**
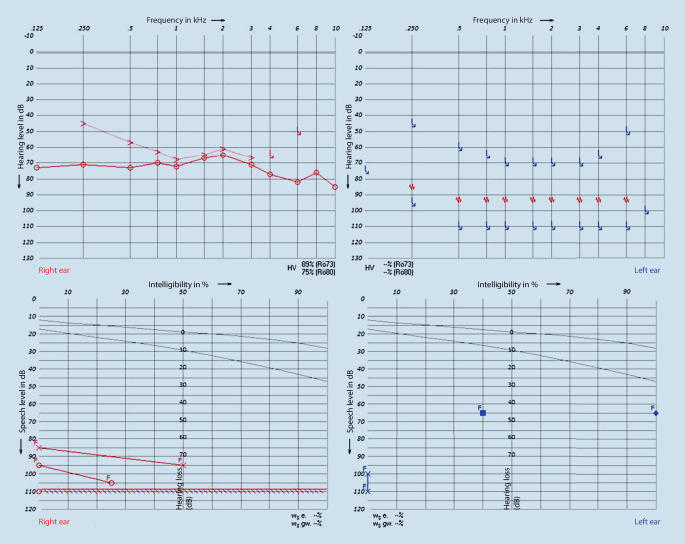
Postoperative pure-tone audiometry and Freiburg Speech Test 4 months after CI surgery on the left side

The patient achieved 100% understanding of numbers and 65% understanding of monosyllables at 65 dB with CI on the left side 4 months after surgery. On the right side, hearing was unchanged (Fig. 3).

## Discussion

The COVID-19 disease, first described in December 2019, is caused by infection with SARS-CoV2, a novel RNA beta-coronavirus. The long-term sequelae and the effect of a COVID-19 infection on the sensory organs in otorhinolaryngology have hardly been investigated thus far [3].

Otorhinolaryngological symptoms such as sore throat and dyspnea might indicate a COVID-19 infection [2]. At the same time, an impaired sense of smell occurs in COVID-19-infected patients. Bocksberger et al. reported that an impaired sense of smell is not associated with rhinitis symptoms in the majority of cases, which may point to a neurogenic cause [4].

Little is known particularly about the influence of a COVID-19 infection on hearing and the labyrinthine system. Mustafa reported significantly impaired high-tone thresholds and reduced OAE amplitudes with respect to the hearing of COVID-19 patients compared with non-infected individuals [9].

As an etiological factor of the pronounced, irreversible hearing loss in the previously normal-hearing 38-year-old patient, a SARS-CoV2-associated sequela in the framework of a COVID-19 infection was considered. Various hypotheses for the pathomechanism of impaired hearing as part of a COVID-19 infection are possible and will be considered here.

In addition to a systemic infection as part of septicemia, a local infection such as labyrinthitis may have resulted in inner-ear damage in our patient. Influenza viruses can cause an infection of the perilymph spaces; accordingly, SARS-CoV2 infection could also cause hearing loss and affect the vestibular organ by direct intralabyrinthine viral manifestation. It is suspected that the blood–labyrinth barrier is disrupted at the peak of infection. The antigen–antibody complex or immune response to viral infection may cause sensory hearing loss in the period of abatement after an acute COVID-19 infection, which occurs within 3 or 4 weeks [10]. Furthermore, labyrinthitis can lead to fibrosis and ossification of the cochlea, which may complicate or even prevent implantation of a CI electrode carrier. Therefore, prompt implantation before the obliteration process is advised [8]. The presence of rotatory vertigo and under-excitability of the left vestibular organ was suggestive of labyrinthitis in our patient.

To verify this hypothesis, inner ear fluid was obtained intraoperatively from the patient for virological examination; no viral RNA was identified. At the same time, the patient’s serum showed a highly positive IgG value (8.4/positive) against SARS-CoV2. The patient had thus already formed antibodies, which might explain why no viral RNA was found in the inner ear fluid at the time of the operation.

Another explanation for the pathomechanism could be the thromboembolic potential of the virus. Al-Ani et al. reported elevated D‑dimer levels in COVID-19 patients with serious respiratory courses. It is assumed that the SARS-CoV2 virus promotes thrombotic complications [1, 9]. Hearing loss could thus arise as a thromboembolic complication due to cochlear perfusion impairment caused by a SARS-CoV2 infection.

It should be considered that increased vasculitis is observed in SARS-CoV2 infections, which are associated with an elevated risk of stroke [5]. The brain MRI study of our patient showed no ischemic change, and thus vasculitis was not assumed in this case.

Herold et al. reported elevated cytokine and interleukin (IL)-6 values in COVID-19 patients. It is currently being examined whether IL‑6 is only a marker of disease activity or perhaps a central factor in the inflammation process COVID-19 infections [7]. Such a possible cytokine storm could result in functional impairment and organ failure, including of the labyrinth with resultant deafness. The IL‑6 laboratory values of our patient were in the normal range during the COVID-19 infection.

It should also be taken into account that hearing loss caused by a COVID-19 viral infection might be brought about by an infection of the nerve sheaths and brain regions. A neuroinvasive potential similar to that already known for coronaviruses is suspected for SARS-CoV2 [3]. Regarding the cause of the loss of smell, it is assumed that the virus enters the rhinencephalon directly via the filiae olfactoriae and then spreads to other regions of the brain. Bocksberger et al. assumed a neurogenic cause of the changes in the olfactory sense caused by COVID-19 infection [4]. Local inflammation of individual brain regions, elicited by the virus, could also cause a central loss of hearing, as is known for an impaired sense of smell. The effect of the SARS-CoV2 virus on the auditory system has not yet been investigated [5]. Damage to auditory neuronal structures in our patient is not likely due to very good hearing rehabilitation with the CI. The present diagnostic tests also showed damage to the patient’s hair cells in the postinfectious absence of OAE. As a differential diagnosis, cochlea disorder due to ototoxic medication (furosemide) must be taken into account, whereby such hearing loss is rarely irreversible, especially with rapid and long-term i.v. medication [6]. Furosemide was immediately withdrawn after 2 days when hypacusis was reported, but there was no improvement in the hearing loss. Rare hearing loss, but not deafness, has been described in association with ECMO therapy [11].

Other reasons that may be considered among the causes of sensory-neural hearing loss—such as acoustic trauma, noise-induced hearing loss, head trauma, labyrinth concussion, diabetes mellitus, hyperlipidemia, presbycusis, disorders such as Meniere’s disease, and otosclerosis—could be excluded from the patient’s medical history [10].

Ultimately, the cause of the hearing loss associated with the COVID-19 infection in this patient has not yet been unequivocally determined.

## Practical conclusion

- An effect of a COVID-19 infection on the auditory system is possible.
- Audiometric testing of COVID-19-infected patients is urgently recommended.

## Declarations

For this article no studies with human participants or animals were performed by any of the authors. All studies performed were in accordance with the ethical standards indicated in each case. Additional written informed consent was obtained from all individual participants or their legal representatives for whom identifying information is included in this article.

The supplement containing this article is not sponsored by industry.
